# Momentum considerations inside near-zero index materials

**DOI:** 10.1038/s41377-022-00790-z

**Published:** 2022-04-25

**Authors:** Michaël Lobet, Iñigo Liberal, Larissa Vertchenko, Andrei V. Lavrinenko, Nader Engheta, Eric Mazur

**Affiliations:** 1 John A. Paulson School of Engineering and Applied Sciences, Harvard University, 9 Oxford Street, Cambridge, MA 02138 USA; 2 Department of Physics and Namur Institute of Structured Materials, University of Namur, Rue de Bruxelles 51, 5000 Namur, Belgium; 3 Electrical and Electronic Engineering Department, Universidad Pública de Navarra, Campus Arrosadía, Pamplona, 31006 Spain; 4 Department of Photonics Engineering, Technical University of Denmark, Ørsteds Plads 345A, DK-2800 Kgs Lyngby, Denmark; 5 Department of Electrical and Systems Engineering, University of Pennsylvania, Philadelphia, PA 19104 USA

**Keywords:** Metamaterials, Optics and photonics

## Abstract

Near-zero index (NZI) materials, i.e., materials having a phase refractive index close to zero, are known to enhance or inhibit light-matter interactions. Most theoretical derivations of fundamental radiative processes rely on energetic considerations and detailed balance equations, but not on momentum considerations. Because momentum exchange should also be incorporated into theoretical models, we investigate momentum inside the three categories of NZI materials, i.e., inside epsilon-and-mu-near-zero (EMNZ), epsilon-near-zero (ENZ) and mu-near-zero (MNZ) materials. In the context of Abraham–Minkowski debate in dispersive materials, we show that Minkowski-canonical momentum of light is zero inside all categories of NZI materials while Abraham-kinetic momentum of light is zero in ENZ and MNZ materials but nonzero inside EMNZ materials. We theoretically demonstrate that momentum recoil, transfer momentum from the field to the atom and Doppler shift are inhibited in NZI materials. Fundamental radiative processes inhibition is also explained due to those momentum considerations inside three-dimensional NZI materials. Absence of diffraction pattern in slits experiments is seen as a consequence of zero Minkowski momentum. Lastly, consequence on Heisenberg inequality, microscopy applications and on the canonical momentum as generator of translations are discussed. Those findings are appealing for a better understanding of fundamental light-matter interactions at the nanoscale as well as for lasing applications.

## Abstract (web summary)

Light momentum inside near-zero refractive index materials is analyzed using Abraham–Minkowski discussion. It explains the absence of fundamental radiative processes, of diffraction patterns or Doppler effect inside such materials.

## Graphical abstract

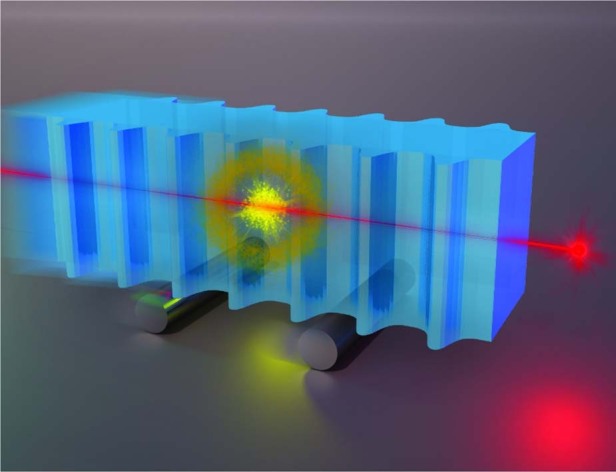

## Introduction

In his seminal papers introducing fundamental radiative processes^1,2^, Einstein noted that, while the description of the interaction between light and matter typically only take into account energy exchange, energy and momentum are directly connected to each other, and momentum exchange is equally important. A consequence of Einstein’s theory of radiation is that the absorption/emission of a quantum of energy *ħω* is accompanied by a momentum transfer $\hbar \omega /c = \hbar k$ between the field and the atom, with *ħ* the reduced Planck constant and *k* being the wavevector. When an atom absorbs radiation, the momentum transfer is in the direction of propagation of the photon, while for emission the transfer is in the opposite direction, inducing a recoil of the atom. In a medium, the momentum of electromagnetic radiation (“electromagnetic momentum”) depends on the refractive index. However, there has been a long-standing debate concerning the dependence of the electromagnetic momentum on the refractive index depending on whether one uses the Minkowski^3^ or Abraham^4,5^ formulation of the electromagnetic momentum. The electromagnetic momentum density in the Abraham (**g**_*A*_) and Minkowski (**g**_*M*_) forms are

1 $${{{\mathbf{g}}}}_A = {{{\mathbf{E}}}} \times {{{\mathbf{H}}}}/c^2$$

and

2 $${{{\mathbf{g}}}}_M = {{{\mathbf{D}}}} \times {{{\mathbf{B}}}}$$

respectively^6,7^. These formulations yield the following two expressions for the magnitude of the electromagnetic momentum in a dispersive medium:

3 $$p_A = \frac{{\hbar \omega }}{{n_g(\omega )c}}$$

and

4 $$p_M = n_\varphi (\omega )\frac{{\hbar \omega }}{c}$$

for the Abraham momentum *p*_*A*_ and the Minkowski momentum *p*_*M*_, respectively, and where $n_\varphi (\omega ) = \sqrt {\varepsilon (\omega )\mu (\omega )}$ is the phase refractive index and $n_g = c\left( {\frac{{d\omega }}{{dk}}} \right)^{ - 1}$ the group refractive index.

Note that in a non-dispersive medium, $n_\varphi = n_g = n$ and consequently $p_M = n\frac{{\hbar \omega }}{c}$ and $p_A = \frac{{\hbar \omega }}{{nc}}$. The difference between those two expressions for the electromagnetic momentum is at the heart of Abraham–Minkowski debate. Some experiments appear to support the Minkowski formulation^8–10^, while others support the Abraham formulation^11–13^.

A resolution of this long-lasting dilemma was recently proposed^14,15^ by attributing the difference between the Abraham and Minkowski momenta of light to the duality of light and matter^6^. For a particle, the classical (particle) momentum is given by the kinetic momentum, defined as $p_{kin} = p_A$. On the other hand, the canonical momentum, $p_C = h/\lambda$, embodies the wavelike nature of the particle. It was shown that in any light-matter interaction^14,15^, the total momentum—a conserved quantity—is given by the sum of the kinetic momentum of the particle and the Abraham momentum of the light and is equal to the sum of the canonical momentum of the particle and the Minkowski momentum of the light:

5 $$p_{{\rm{kin}}}^{{\rm{medium}}} + p_A = p_C^{{\rm{medium}}} + p_M$$

One could therefore call the Abraham momentum the “kinetic momentum of the light” and the Minkowski momentum the “canonical momentum of the light”^14,15^. In other words, the Abraham momentum comes into play when considering the particle nature of light and the Minkowski momentum when considering the wavelike nature of light^16^. In this paper we re-examine the difference between these two formulations in the extreme case that the index of refraction of the medium approaches zero.

## Results

### Momentum inside near-zero index materials

In the past decade materials with near-zero refractive index have received a lot of attention because of their unusual optical properties, such as supercoupling^17,18^, enhanced nonlinearities^19–22^ and fluorescence^23–25^, control of dipole-dipole interactions^26,27^, geometry-invariant resonant cavities^28^, photonic doping^29^ and propagation of the light power flow akin to ideal fluids^30^. The refractive index of a material is near zero when at least one of the two constitutive parameters of the refractive index — the relative electric permittivity *ε*(*ω*) or the relative magnetic permeability *μ*(*ω*) — is close to zero^31,32^. Near-zero index materials (NZI materials) fall into three categories: epsilon-near-zero (ENZ) materials where *ε* approaches zero with nonzero *μ*;^17,33^ mu-near-zero (MNZ) materials with *μ* approaching zero with nonzero permittivity *ε*^34^; or epsilon-and-mu-near-zero (EMNZ) media where both *ε* and *μ* approach zero simultaneously^28,32,35–37^.

### Phase and group indices inside NZI materials

At the zero-index frequency in a NZI materials, the phase index is zero, but it is important to note that the group index for an infinite, lossless material depends on the NZI materials category^31,38^.

6 $$n_g\left( {\omega = \omega _Z} \right) = \left\{ {\begin{array}{*{20}{l}} \infty \hfill & {{{{\mathrm{ENZ \& MNZ}}}}\,{{{\mathrm{materials}}}}} \hfill \\ {\omega _Z\partial _\omega n_\varphi \left( {\omega _Z} \right)} \hfill & {{{{\mathrm{EMNZ}}}}\;{{{\mathrm{materials}}}}} \hfill \end{array}} \right.{}$$

Consequently, the group velocity *v*_*g*_ is zero at the zero-index frequency in unbounded ENZ/MNZ materials^39^ but nonzero for EMNZ materials: $\left( {v_g(\omega = \omega _Z) = c/\omega _Z\partial _\omega n\left( \omega \right)} \right)$^38^. It should remain positive in low loss material, as imposed by causality. Note that, despite having a near-zero group velocity, energy can be transmitted through a finite size ENZ(MNZ) sample^40^. An exhaustive discussion on group and energy velocities inside infinite NZI materials sample is provided in Supplementary materials.

### Minkowski momentum inside NZI materials

**Fig. 1:**
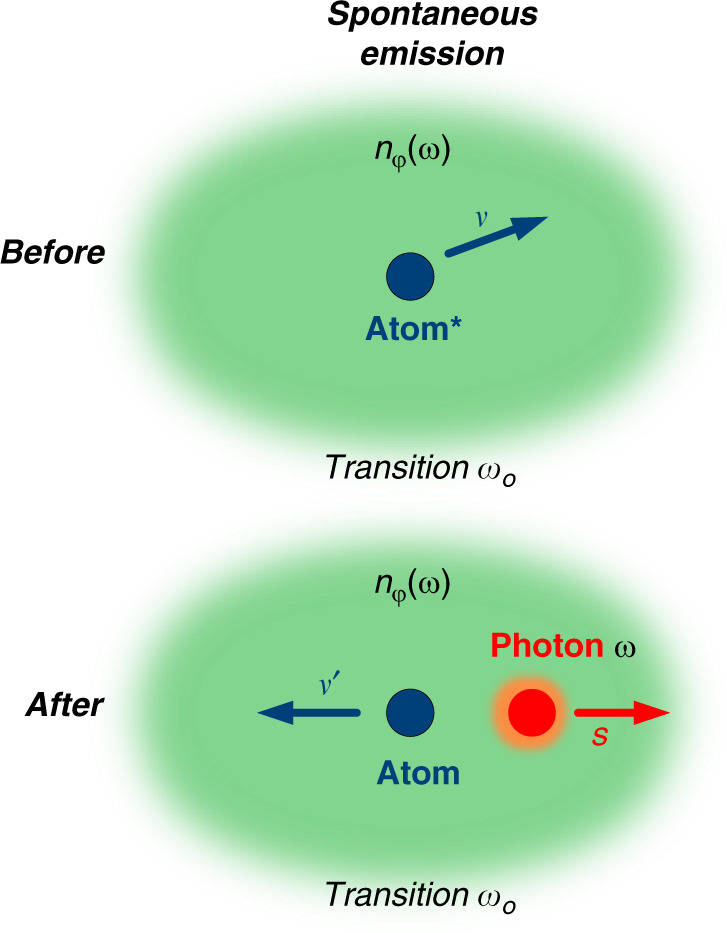
Schematic of the spontaneous emission process inside NZI materials of refractive index *n*_*φ*_(*ω*) (green background). An atom (blue) moves around at a velocity *v* (*v*′) before (after) spontaneous emission process. A photon (red) is being emitted after the excited atom relaxed in its ground state, in direction ***s***

Because of the zero phase refractive index the Minkowski momentum—the canonical momentum of light—is zero for all NZI materials categories: $p_C = p_M = 0$ see Eq. (4). Another way to show that the Minkowski momentum is zero involves applying the de Broglie relationship, $p_C = \frac{h}{\lambda }$, inside an NZI materials, which yields $p_C = 0$, because the effective wavelength $\lambda = \lambda _0/n_\varphi$ tends to infinity inside NZI materials, where *λ*_0_ is the vacuum wavelength^31^. Another consequence is that no momentum is imparted by the photon to the material inside a NZI materials. This point can be clarified using the example of the Doppler shift that occurs during spontaneous emission of radiation. Let us suppose an emitting atom of mass *m*, with a transition frequency *ω*_0_, an initial velocity ***v*** and a final velocity ***v***′ after emitting a photon of frequency *ω* (Fig. 1)^14,15^.

In the non-relativistic approximation^41^, conservation of energy for the spontaneous emission process implies

7 $$\frac{{mv^2}}{2} + \hbar \omega _0 = \frac{{mv\prime ^2}}{2} + \hbar \omega$$

while the conservation of linear momentum can be expressed as

8 $$m{{{\boldsymbol{v}}}} = m{{{\boldsymbol{v}}}}\prime + \hbar {{{\boldsymbol{k}}}}$$

with ${{{\boldsymbol{k}}}} = \left[ {n_\varphi (\omega )\frac{\omega }{c}} \right]{{{\boldsymbol{s}}}}$, **s** being an unit vector pointing in the direction of the emitted photon and $- \hbar {{{\boldsymbol{k}}}}$ is the recoil momentum of the atom. As is well known from classical physics, the frequency of the emitted light, as it appears to the moving atom, is increased due to the Doppler shift. The Doppler shift formula can be deduced as^42,43^

9 $$\omega = \omega _0\left[ {1 + \frac{{n_\varphi (\omega )}}{c}v{\rm{cos}}\theta } \right]$$

where *θ* is the angle between ***v*** and **s**. In general, $\hbar {{{\boldsymbol{k}}}}$ is not solely the momentum of the emitted photon, but corresponds to the total momentum transferred from the atom to both the emitted photon and the medium^6^. The recoil of an emitter in a dispersive dielectric can be calculated either by using a macroscopic theory of spontaneous emission (considering the source atom as a two-level atom with a transition dipole ***d***) or by using field quantization in the dielectric^6^. Both approaches yield to the conclusion that the recoil momentum is the canonical momentum:

10 $$p_C = n_\varphi (\omega _0)\frac{{\hbar \omega _0}}{c}$$

Consequently, the recoil momentum vanishes inside NZI materials. Moreover, the Doppler shift perceived by the atom also vanishes as the phase refractive index goes to zero (Eq. (9)). This extinction of the Doppler shift can be understood as a continuous transition between inverse Doppler effect occurring in negative index materials^44–46^ and regular Doppler effect in positive index materials. Intuitively, inside NZI materials there is no phase difference, all parts of the material are tight to the same phase since the phase velocity is infinite. The compression or expansion of the wave fronts is not possible and the Doppler effect consequently vanishes. It should be noted here that local field corrections have no effect on the inhibition of the recoil momentum of the atom. Furthermore, a similar analysis can be done for deriving the recoil momentum in stimulated emission or in absorption processes^10^ and will yield to the same conclusions in NZI materials. The absence of recoil momentum as a consequence of zero Minkowski momentum provides another way to understand inhibition of fundamental radiative processes inside three-dimensional NZI materials^38^. NZI materials forbid momentum exchange and the atom to recoil, in absorption and emissions processes. This can be seen as an environmental effect. This conclusion is totally consistent with our previous findings based solely on energy and detailed balance considerations^38^. Energy and momentum considerations are now treated on equal footing for the question of fundamental radiative processes as Einstein originally suggested^1,2^. In summary, once wave aspects are dominating, marked in equations by the presence of the phase refractive index or the canonical momentum, related phenomena are inhibited inside NZI materials.

### Abraham momentum inside NZI materials

**Fig. 2:**
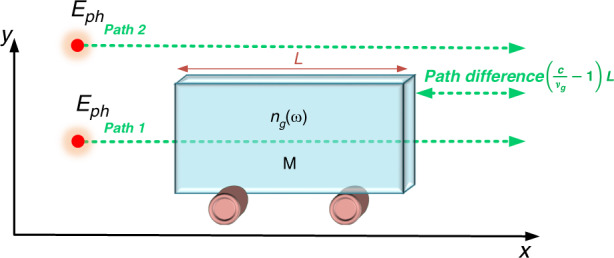
Schematic of Balazs gedanken experiment inspired by^48,49^. The photon can either take path 1 inside the slab of mass *M*, length *L* and group index *n*_*g*_(*ω*) or follow path 2 in free space. The slab is supposed to move freely, without friction in the *x* direction

Consequences of near-zero refractive index on momentum considerations are different in particle-oriented experiments compared to wave-oriented experiments. Therefore, let us discuss one important particle-oriented experiment, the Balazs gedanken experiment^47^, applied to NZI materials. A detailed analysis of this gedanken experiment can be found in^15,48,49^ and is partly reproduced in Supplementary materials. A photon propagates inside a transparent dielectric slab of length *L*, having a group refractive index $n_g(\omega )$ (Fig. 2). The slab can move without any friction along the *x* axis and is supposed to be initially at rest (*v* = 0). The photon propagates in the *x* direction, enters the slab from the left facet and exits from the right facet. We suppose no losses due to absorption or scattering. The photon of energy *ħω* propagates at velocity *c* out of the slab (path 2), but propagates at the group velocity *v*_*g*_ within the slab (path 1). By applying energy and momentum conservation laws, we can calculate the momentum gained by the slab *p*_slab_ as well as the displacement of the slab Δ*x* due to the propagation of the photon following path 1. From there, we can deduce the momentum of the photon inside the slab, which reduces to the Abraham momentum (details in Supplementary materials) as given by Eq. (3). We remind here that the group index is the relevant one for the Abraham momentum and differs between ENZ/MNZ and EMNZ categories.

For ENZ/MNZ media, the group index is infinite (Eq. (6)), and the group velocity is consequently zero^39^. Therefore, the Abraham momentum is also zero (Eq. (3)). For a sufficiently large lossless ENZ/MNZ slabs, the photon is completely reflected, and it bounces back at the material interface, communicating a forward momentum of $2\frac{{\hbar \omega }}{c}$ to an unbounded lossless ENZ/MNZ slab. However, inside an EMNZ medium, the group index is nonzero and equal to $\omega _Z\partial _\omega n_\varphi \left( {\omega _Z} \right)$ (Eq. (6)). Therefore, propagation is allowed inside the slab that is displaced by a quantity ${{\Delta }}x_{EMNZ} = \left( {\omega _Z\partial _\omega n_\varphi \left( {\omega _Z} \right) - 1} \right)L\frac{{\hbar \omega }}{{Mc^2}}$ (details in Supplementary materials). The slab acquires a momentum given by $p_{{\rm{slab}}} = \left( {1 - \frac{1}{{\omega _Z\partial _\omega n_\varphi \left( {\omega _Z} \right)}}} \right)\frac{{\hbar \omega }}{c}$. Those considerations point a difference between EMNZ materials and photonic crystals. Experimental realization of EMNZ materials are photonic crystals showing a linear band dispersion around ${{\Gamma }} = 0$, a crossing at the so-called Dirac point and a vanishing density of states at this point^32,36,50^. Even if spontaneous emission is forbidden inside such EMNZ photonic crystal^38^, propagation within EMNZ material is allowed. This is not the case for classical photonic crystals around their photonic bandgap^51^. Consequently, photonic crystals with EMNZ properties allow both propagation of electromagnetic radiation and inhibition of spontaneous emission simultaneously, which are interesting for lasing platforms^52–54^.

### Consequence of zero Minkowski momentum on diffraction

Considerations on momentum inside NZI materials also have consequences on diffraction phenomena, e.g., on slit experiments inside dispersive media. Young double-slit experiments immersed inside a dielectric liquid can be found in literature, e.g.,^12,55,56^. Recently, double-slit experiments were performed inside one of the NZI materials category, ENZ materials^57^.

**Fig. 3:**
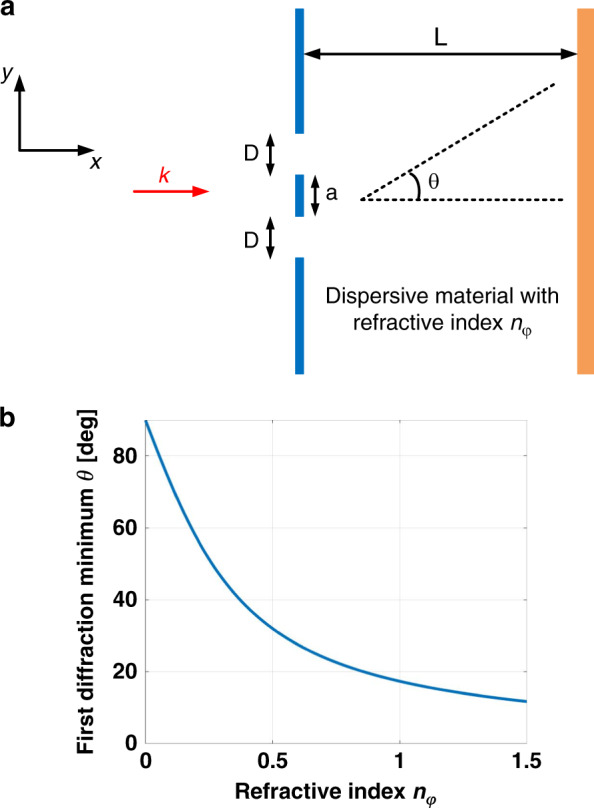
Young double-slit experiment within dispersive material. **a** EM wave with ***k*** wavevector is sent on a double-slit with slit width *D* at a distance *L* from an observing screen. The distance between the slits is *a*. **b** First diffraction minimum as a function of refractive index. Wavelength is set to 500 nm for a separation width of *a* = 800 nm

Here, we consider a double-slit experiment, with a slit width *D*, separated by a distance *a* (Fig. 3a). The distance between the double-slit and the observing screen is denoted as *L* and the whole system, including the double-slit and the screen, are embedded inside a dispersive medium of refractive index *n*_*φ*_. If *θ* denotes the angle between the forward *x* direction and the direction of the first diffraction minimum, diffraction theory gives

11 $${\rm{tan}}(\theta ) = \frac{{\lambda _0}}{{2a|n_\varphi |}}$$

if $L \,> > \, a$. In positive refractive index materials, the diffraction angle *θ* is consequently lowered by a factor $|n_\varphi |$ (Fig. 3b, for $n_\varphi\, > \,1$) while the corresponding canonical momentum *p*_*x*_ is increased by the same factor. In NZI materials (Fig. 3b, for $n_\varphi \,<\, 1$), the opposite situation occurs: the first diffraction minimum moves away from the *x* axis as *n*_*φ*_ decreases, while the canonical momentum tends towards zero. The localization in the momentum space imposes a delocalization in the position space, as a consequence of Heisenberg inequalities. Moreover, the intensity distribution on the screen of this double-slit experiment follows

12 $$I(y) = \frac{{I_0}}{2}\frac{{{\rm{sin}}^2\left( {\frac{{\pi Dy}}{{\lambda L}}} \right)}}{{\left( {\frac{{\pi Dy}}{{\lambda L}}} \right)}}\left[ {1 + {\rm{cos}}\left( {\frac{{2\pi ay}}{{\lambda L}}} \right)} \right]$$

with *I*_0_ being the intensity of the incident wave and *y* the vertical position on the screen. As the refractive index goes to zero, the effective wavelength *λ* inside the medium goes to infinity, therefore the cos term tends to one. Recalling that the sinc function is equal to one at the zero-limit, the intensity on the screen appears to be constant, i.e., $I(y) \to I_0$. This calculation confirms that the first order diffraction minimum is removed to infinity and that diffraction effects are reduced in NZI materials. Same conclusions hold for single-slit experiments. It is interesting to note that regarding single-slit experiments, the suppression of diffraction pattern inside NZI materials is nothing but a consequence of Babinet’s principle, i.e., diffraction pattern of a slit or of a rectangular object should be similar. Inside NZI materials, no scattering of objects can be identified rendering them invisible^50^. Cloaking corresponds to an infinite incertitude on the position of the invisible object, which can be reached using NZI materials as discussed above.

**Fig. 4:**
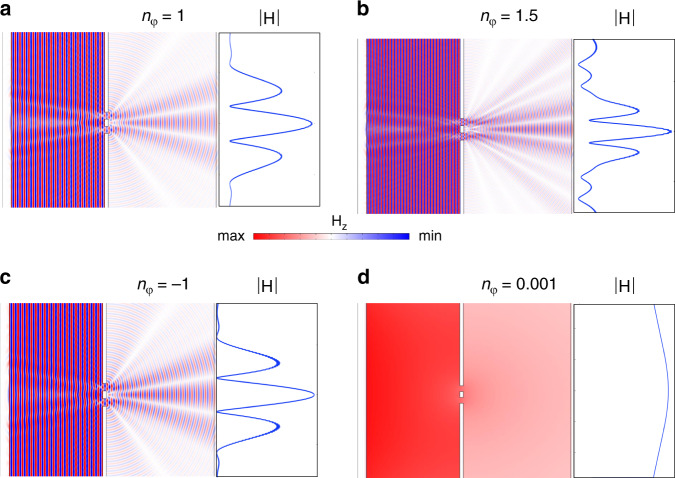
Young double-slit experiment within different dispersive materials. Left: $|H_z|$ field maps, right: $|H|$ profile on the observing screen. **a** Air ($\varepsilon = \mu = 1$,$n_\varphi = 1$), **b** dielectric material such as glass ($\varepsilon = 2.25,\mu = 1$,$n_\varphi = 1.5$), **c** a negative refractive index material ($\varepsilon = - 1,\mu = - 1$,$n_\varphi = - 1$) and **d** an ENZ material ($\varepsilon = 1 \times 10^{ - 6}$, $\mu = 1$ and $n = 0.001$). Wavelength is set to 500 nm for a separation width of 800 nm

The above theoretical considerations are verified by full-wave simulations (see Materials and methods) within four different materials: (a) air ($\varepsilon = \mu = 1$, $n_\varphi = 1$), (b) dielectric material such as glass ($\varepsilon = 2.25,\mu = 1$, $n_\varphi = 1.5$), (c) a negative refractive index material ($\varepsilon = - 1,\mu = - 1$, $n_\varphi = - 1$) and (d) an ENZ material ($\varepsilon = 1 \times 10^{ - 6}$, $\mu = 1$ and $n = 0.001$). As we can observe on Fig. 4, the diffraction patterns (here, the *H* field component) gets compressed inside dielectric material with refractive index higher than air (Fig. 4b), while no diffraction pattern appears within ENZ medium (Fig. 4d). The corresponding intensity profile on the screen and the direction of the first diffraction minimum are consistent with Eqs. (11) and (12).

Finally, it is interesting to point that diffraction patterns are not influenced by the sign of the refractive index, but only by its absolute value (Fig. 4c). Consequently, the NZI materials scenario is an extreme case for diffraction theory as presented above.

### Consequence on Heisenberg inequality and microscopy

Let us evaluate how the momentum considerations derived above impact the Heisenberg position-momentum inequality and its implication in microscopy. We have shown that the canonical momentum *p*_*C*_ reaches zero in NZI materials (Eq. (10)). The exact knowledge of the momentum value leaves it with no uncertainty, i.e., Δ*p* = 0. This is based on the assumption $n_\varphi \left( x \right) = n_\varphi$ (constant real RI), which leads to ${{\Delta }}p = \left| {n_\varphi } \right|{{\Delta }}p_0$. Since $n_\varphi = 0,{{{\mathrm{we}}}}\,{{{\mathrm{have}}}}\,{{\Delta }}p = 0$. Position and the momentum should satisfy the Heisenberg uncertainty ${{\Delta }}x{{\Delta }}p \ge h,$ exact knowledge of the zero canonical momentum inside NZI media, i.e., localization in the *k* space, imposes an infinite uncertainty in the position space, i.e., the particle is delocalized and its wavelength being infinite. To further confirm this insight and to find its implications on microscopy, we recall that the numerical aperture of the microscope is $NA = |n_\varphi |{\rm{sin}}{{\Phi }}$ with Φ being the semi-aperture angle. The smallest distance between two resolved points, i.e., the resolution, is equal to ${{\Delta }}x = \frac{{\lambda _0}}{{2NA}}$, referring to the well-known Abbe diffraction limit^58^. Because *NA* = 0 in NZI media, Δ*x* = ∞. If one could realize a microscope inside an NZI material, the resolution of such a microscope would be very poor and unsuitable for any imaging purpose. Consequently, being inside an NZI materials would lead to an infinite uncertainty on position and zero uncertainty on momentum. Conceptually, this implies that since the resolution is poor and no correct image can be formed, an object of any shape and material can be “hidden” in a NZI material.

The above discussion is also consistent with the angular spectrum representation of fields in the NZI media. In writing the general expression ${{{\boldsymbol{E}}}}\left( {x,y,z} \right) = {\int} {{\int}_{ - \infty }^{ + \infty } {{{{\hat{\boldsymbol E}}}}\left( {k_x,k_y;0} \right)} } \,e^{i\left[ {k_xx + k_yy \pm k_zz} \right]}dk_xdk_y$ with $k = \sqrt {k_x^2 + k_y^2 + k_z^2} = n_\varphi \left( {\frac{\omega }{c}} \right) = n_\varphi k_0$, the 2D Fourier spectrum ${{{\hat{\boldsymbol E}}}}$ evolves along the *z* axis as ${{{\hat{\boldsymbol E}}}}\left( {k_x,k_y;z} \right) = {{{\hat{\boldsymbol E}}}}\left( {k_x,k_y;0} \right)e^{ \pm ik_zz}$. Since $k = n_\varphi k_0 = 0$ in NZI media, the wavenumber component *k*_*z*_ is imaginary, and therefore all those spatial frequencies, containing the information on high spatial variations of an object that would be inside the NZI materials, are filtered out.

### Canonical momentum as the generator of translations

Let us address the decoupling of electric and magnetic fields in a plane-wave from a vector potential perspective. The canonical moment is the generator of translation^14,15^ represented by the unitary transformation

$${{{\mathrm{e}}}}^{\frac{{{{{\mathrm{ib}}}}}}{\hbar }{{{\mathrm{p}}}}_{{{\mathrm{c}}}}}f\left( z \right){{{\mathrm{e}}}}^{ - \frac{{{{{\mathrm{ib}}}}}}{\hbar }{{{\mathrm{p}}}}_{{{\mathrm{c}}}}} = f(z + b)$$

where *b* is a constant. Inside NZI material, since p_c_ = 0, the above expression leads to constant *f*(*z*) inside the material. A direct example of such *f*(*z*) function is the phase inside NZI materials.

Moreover, the Minkowski momentum is the canonical momentum of the electromagnetic field. Therefore, it generates translations of plane-wave modes according to^15^

$${{{\mathrm{e}}}}^{ - \frac{{{{\mathrm{i}}}}}{\hbar }{{{\boldsymbol{b}}}} \cdot {\int} {dVg_{{\rm{Min}}}} }{{{\boldsymbol{A}}}}({{{\boldsymbol{r}}}}){{{\mathrm{e}}}}^{\frac{{{{\mathrm{i}}}}}{\hbar }{{{\boldsymbol{b}}}} \cdot {\int} {dVg_{{\rm{Min}}}} } = {{{\boldsymbol{A}}}}({{{\boldsymbol{r}}}} + {{{\boldsymbol{b}}}})$$

with ***A*** the (electric) vector potential in the Coulomb gauge and ***b*** a constant vector. Here again inside NZI materials, we obtain a constant vector potential. Consequently, we obtain a spatially constant (but temporally oscillating) electric field ${{{\boldsymbol{E}}}}_{{{\boldsymbol{A}}}} = - i\omega {{{\boldsymbol{A}}}}$. As for the magnetic field we have ${{{\boldsymbol{H}}}}_{{{\boldsymbol{A}}}} = \frac{1}{\mu }\nabla \times {{{\boldsymbol{A}}}}$. If we are in an ENZ medium (where *ε* is zero, but *μ* is not), we obtain zero magnetic field. This is consistent with the fact that in an ENZ medium, the intrinsic impedance for a uniform plane wave is infinite. Similarly, we can write for the magnetic vector potential^59^

$${{{\mathrm{e}}}}^{ - \frac{{{{\mathrm{i}}}}}{\hbar }{{{\boldsymbol{b}}}} \cdot {\int} {dVg_{{\rm{Min}}}} }{{{\boldsymbol{F}}}}({{{\boldsymbol{r}}}}){{{\mathrm{e}}}}^{\frac{{{{\mathrm{i}}}}}{\hbar }{{{\boldsymbol{b}}}} \cdot {\int} {dVg_{{\rm{Min}}}} } = {{{\boldsymbol{F}}}}({{{\boldsymbol{r}}}} + {{{\boldsymbol{b}}}})$$

with the associated magnetic field ${{{\boldsymbol{H}}}}_{{{\boldsymbol{F}}}} = - i\omega {{{\boldsymbol{F}}}}$ and electric field ${{{\boldsymbol{E}}}}_{{{\boldsymbol{F}}}} = - \frac{1}{\varepsilon }\nabla \times {{{\boldsymbol{F}}}}$. Here, ***H***_***F***_ remains spatially constant (but temporally oscillating) in NZI materials, while ***E***_***F***_ vanishes in MNZ materials, consistent with the fact that in MNZ media, the intrinsic impedance for the uniform plane wave is 0. It can be noted that ***H***_***A***_ and ***E***_***F***_ are irrotational in ENZ and MNZ materials, respectively^30^.

## Discussion

Momentum considerations inside dispersive near-zero refractive index materials are theoretically worked out using the recent resolution of the Abraham–Minkowski debate^14,15^. We evidenced that canonical-Minkowski momentum is identically zero inside NZI materials. This inhibits wave-related phenomena inside NZI materials. The Doppler shift perceived by the moving atom inside NZI materials is canceled. No recoil momentum occurs inside such an unbounded lossless material. The dispersive material forbids the atom to recoil both in emission or absorption processes, leading to an absence of momentum exchange inside NZI materials. Fundamental radiative processes are inhibited inside three-dimensional NZI materials accordingly and this conclusion is consistent with the one derived using solely energetic considerations^38^. Energy and momentum are now treated on an equal footing regarding fundamental radiative processes inside NZI materials as Einstein suggested in seminal works^1,2^. Absence of diffraction also appears as consequence of zero canonical momentum within NZI materials. Consequences of zero canonical-Minkowski momentum on Heisenberg inequality, on microscopy as well as on potential vectors are also discussed. Nevertheless, for experiments where the corpuscular nature of light is probed, the Abraham momentum is linked to the group refractive index and therefore a distinction should be made according to the NZI materials category. Unbounded lossless ENZ/MNZ materials forbid direct propagation with zero kinetic-Abraham momentum, while bounded EMNZ allows direct propagation and nonzero kinetic-Abraham momentum. EMNZ-based photonic crystals can then be considered as specific materials allowing both light propagation but inhibiting spontaneous emission. This property is appealing for controlling fundamental radiative processes at the nanoscale as well for lasing perspectives.

## Materials and methods

Most of the presented works rely on analytical derivations based on the reported literature.

The double-slit analysis was conducted by numerical simulations performed by the commercially available software COMSOL (version 5.6). We used the “Electromagnetic waves, frequency domain” module to analyze the *H*_*z*_ field profiles at a cut-line placed at 10 µm away from the slits. An active port generates the normally incident field with wavelength set to 500 nm. Perfectly matched layers are used as boundary conditions. The estimated calculation time is a few minutes per system.

Supplementary information accompanies the manuscript on the *Light: Science & Applications* website (http://www.nature.com/lsa).

## Supplementary information

Supplementary material
