# Supplementary material for: Momentum considerations inside near-zero index materials
Source: Light Sci Appl. 2022 Apr 25;11:110. doi: 10.1038/s41377-022-00790-z (PMC9039083; doi:10.1038/s41377-022-00790-z)
Supplement: Supplementary file 1 — Supplementary material [file 41377_2022_790_MOESM1_ESM.docx]

##### Supplementary Materials of

##### Momentum considerations inside near-zero index materials

Michaël Lobet^1,2,#,*^, Iñigo Liberal^3,#^, Larissa Vertchenko^4,#^, Andrei V Lavrinenko^4^, Nader Engheta^5^, Eric Mazur^1^

^1^John A. Paulson School of Engineering and Applied Sciences, Harvard University, 9 Oxford Street, Cambridge, MA 02138, United States of America

^2^Department of Physics and Namur Institute of Structured Materials, University of Namur, Rue de Bruxelles 51, 5000 Namur, Belgium

^3^Electrical and Electronic Engineering Department, Universidad Pública de Navarra, Campus Arrosadía, Pamplona, 31006 Spain

^4^Department of Photonics Engineering, Technical University of Denmark, Ørsteds Plads 345A, DK-2800 Kgs. Lyngby, Denmark

^5^Department of Electrical and Systems Engineering, University of Pennsylvania, Philadelphia, PA 19104, USA

# These authors contributed equally to this work

*Corresponding author: [michael.lobet@unamur.be](mailto:michael.lobet@unamur.be) (0032473438365)

***Emails of the authors:***

Michaël Lobet: [michael.lobet@unamur.be](mailto:michael.lobet@unamur.be)

Iñigo Liberal: [liberal.inigo@gmail.com](mailto:liberal.inigo@gmail.com)

Larissa Vertchenko: [lariv@fotonik.dtu.dk](mailto:lariv@fotonik.dtu.dk)

Andrei V Lavrinenko : [alav@fotonik.dtu.dk](mailto:alav@fotonik.dtu.dk)

Nader Engheta : [engheta@seas.upenn.edu](mailto:engheta@seas.upenn.edu)

Eric Mazur : [mazur@seas.harvard.edu](mailto:mazur@seas.harvard.edu)

## *Content*

1. Energy density within dispersive and dissipative media and considerations on energy velocity
2. Balazs gedanken experiment
3. Impact of losses on momentum considerations

***

## 1 Energy density within dispersive and dissipative media and considerations on energy velocity

We would like to establish a consistent formalism for the energy density within a dispersive and dissipative material. We will follow the Loudon approach [1], well described by Nunes [2] and generalized to magnetic materials by Ruppin [3].

We analyse the energy density for a wave propagating through a dispersive and dissipative medium with a single resonant frequency. Dissipation can occur with both the E-field and the H-field [3]. A simple model of a damped harmonic oscillator can be used to model dissipation, with $\omega_{0e}$ ($\omega_{0h})$ the resonance frequency of the electric (magnetic) dipole oscillators, $\gamma_{e}$ ($\gamma_{m}$) the damping rate and $\omega_{pe}$ ($\omega_{pm}$) characterizing the strength between the dipole oscillators and the electric (magnetic) field.

The equation of motion of the electric polarization $\mathbf{P}$ in the presence of an oscillating electric field $\mathbf{E}$ can be written in the form

$\ddot{\mathbf{P}}+\gamma_{e}\dot{\mathbf{P}}+\omega_{0e}\mathbf{P}=\varepsilon_{0}\omega_{pe}^{2}\mathbf{E}.$ (13)

Similarly, we can write the equation of motion of the magnetic polarization $\mathbf{M}$ in the presence of an oscillating magnetic field $\mathbf{H}$, in the form

$\ddot{\mathbf{M}}+\gamma_{m}\dot{\mathbf{M}}+\omega_{0m}\mathbf{M}=\mu_{0}F\omega_{pm}^{2}\mathbf{H}.$ (14)

with $F$ a measure of the strength of the interaction between the oscillators and the magnetic field [3].

For harmonic fields, keeping the $e^{-i\omega t}$ time dependence, the electric susceptibility $\chi_{e}$ (without dimensions) is defined by $\mathbf{P}=\varepsilon_{0}\chi_{e}\mathbf{E}$.

The corresponding relative complex permittivity $\tilde{\varepsilon}_{r}(\omega)$ (dimensionless) is defined as

$\tilde{\varepsilon}_{r}(\omega)=\varepsilon_{r}(\omega)'+i\varepsilon_{r}(\omega)''=1+\chi_{e}=1-\frac{\omega_{pe}^{2}}{\omega^{2}-\omega_{0e}^{2}+i\gamma_{e}\omega}$ (15)

$=1-\frac{w_{pe}^{2}(\omega^{2}-\omega_{0e}^{2})}{(\omega^{2}-\omega_{r}^{2})^{2}+\gamma_{e}^{2}\omega^{2}}-i\frac{\omega_{pe}^{2}\gamma_{e}\omega}{(\omega^{2}-\omega_{0e}^{2})^{2}+\gamma_{e}^{2}\omega^{2}}$ (16)

and is related to the permittivity $\tilde{\varepsilon}(\omega)$ [$F/m=\frac{As}{Vm}$] by $\tilde{\varepsilon}(\omega)=\varepsilon_{0}\tilde{\varepsilon}_{r}(\omega)$.

Similarly, the magnetic susceptibility $\chi_{m}$ is defined by $\mathbf{M}=\chi_{m}\mathbf{H}$ and the corresponding relative complex permeability writes

$\tilde{\mu}_{r}(\omega)=\mu_{r}(\omega)'+i\mu_{r}(\omega)''=1+\chi_{m}=1-\frac{F\omega_{pm}^{2}}{\omega^{2}-\omega_{0h}^{2}+i\gamma_{m}\omega}$ (17)

$=1-\frac{w_{pm}^{2}(\omega^{2}-\omega_{0m}^{2})}{(\omega^{2}-\omega_{0m}^{2})^{2}+\gamma_{m}^{2}\omega^{2}}-i\frac{\omega_{pm}^{2}\gamma_{m}\omega}{(\omega^{2}-\omega_{0m}^{2})^{2}+\gamma_{m}^{2}\omega^{2}}$ (18)

and is related to the permeability $\tilde{\mu}(\omega)$ [$H/m]$] by $\tilde{\mu}(\omega)=\mu_{0}\tilde{\mu}_{r}(\omega)$. Consequently, we have the complex refractive index $\tilde{n}=\sqrt{\tilde{\varepsilon}_{r}\tilde{\mu}_{r}}=n'+in''$, the complex wave vector $\tilde{\mathbf{k}}=\mathbf{k}_{\mathbf{0}}\tilde{n}$ and the group velocity $v_{g}=d\omega/dk$. We should note that near an absorptive resonance, the group velocity might be greater than the speed of light (anomalous dispersion) calling for a definition of energy velocity: $v_{E}=\frac{\mathbf{S}}{\overline{w}}$ with $\overline{w}=\overline{w_{e}}+\overline{w_{m}}$ the time-averaged total energy density. Loudon recasted the energy velocity within a lossy material as the ratio of the Poynting vector (time rate of energy flow normal to a unit area) and the total energy density. The above definition leads to the most general form of the total energy density in a medium having both permittivity and permeability dispersive and absorptive. The total energy density $w$ comprises four terms for the stored energy $w_{s}$ and the dissipative energy $w_{d}$ for both the E and H-fields. The time-average of the total energy density $\overline{w}$ writes [2,3]:

$\boxed{\overline{w}=\frac{\varepsilon_{0}}{4}\left( {\varepsilon'}_{r}+\frac{2\omega\varepsilon_{r''}}{\gamma_{e}} \right)|\mathbf{E}|^{2}+\frac{\mu_{0}}{4}\left( \mu_{r'}+\frac{2\omega\mu_{r''}}{\gamma_{m}} \right)|\mathbf{H}|^{2}}.$ (19)

This is the most general form of the energy density in a medium in which both the permittivity and permeability are dispersive and absorptive. The total energy density $w$ is the sum of the electric and magnetic energy densities and each comprise two terms: a *stored energy* term $w_{s}$ and a *dissipative energy* term $w_{d}$ respectively coming from the real and imaginary parts of the permittivity/permeability. It was interestingly described in such a way by Webb in 2012 [4]. We have

$w=w_{s}+w_{d}=w_{se}+w_{sm}+w_{de}+w_{dm}.$ (20)

We can take the free-electron gas limit, $\omega_{0e}\to0$ where the restoring force on the oscillating electrons goes to zero [2]. It is nothing but the Drude model of metals. In this limit, we have $\omega\tau\gg1$, with $\tau$ the scattering time, we have

${\varepsilon'}_{r}(\omega)\to1-\frac{\omega_{pe}^{2}}{\omega^{2}}$ (21)

${\varepsilon''}_{r}(\omega)\to\frac{\gamma_{e}\omega_{pe}^{2}}{\omega^{3}}.$ (22)

From these last two expressions, we have

$\frac{d\varepsilon_{r'}}{d\omega}=\frac{2\omega_{pe}^{2}}{\omega^{3}} \mathrm{and} \omega\frac{d\varepsilon_{r'}}{d\omega}=\frac{2\varepsilon_{r''}\omega}{\gamma_{e}}.$ (23)

Within this limit, Loudon’s approach is equivalent to Brillouin’s approach [2].

Another interesting limit is the one of low losses. It happens when the damping term, controlled by $\gamma_{e}$ (respectively $\gamma_{m}$), goes to zero. According to the equation 16 (eq. 18), the imaginary parts go to zero ($\varepsilon_{r''}\to0$ and $\mu_{r''}\to0$) as expected. Moreover, we can check that for the permittivity [5], we have

$\frac{2\varepsilon_{r''}\omega}{\gamma_{e}}\to\frac{2\omega_{pe}\omega^{2}}{(\omega^{2}-\omega_{0e}^{2})^{2}}$ (24)

and

$\lim_{\gamma_{e}\to0}\frac{d\varepsilon_{r'}}{d\omega}=\frac{2\omega_{pe}\omega}{(\omega^{2}-\omega_{0e}^{2})^{2}}.$ (25)

Consequently, the stored energy density is the limit of eq. 19 for small losses ($\gamma\to0$ and yields

$\lim_{\gamma\to0}\overline{w}=\overline{w_{S}}$ (26)

$=\frac{\varepsilon_{0}}{4}( \varepsilon_{r'}+\omega\frac{d\varepsilon_{r'}}{d\omega}) |\mathbf{E}|^{2}+\frac{\mu_{0}}{4}(\mu_{r'}+\omega\frac{d\mu_{r'}}{d\omega}) |\mathbf{H}|^{2}.$ (27)

The frequency derivatives taking dispersion into account, even if the material does not absorbs intensively. Nunes adds that the dissipated power density [2]

$\frac{\gamma_{e}\omega^{2}}{\varepsilon_{0}\omega_{pe}^{2}}=\varepsilon_{0}{\varepsilon''}_{r}\omega|\mathbf{E}|^{2}$ (28)

also goes to zero in this limit.

If the dissipation is not negligibly small, the term including the dissipative energy density, averaged over an optical cycle is

$\overline{w_{d}}=\frac{\varepsilon_{0}}{2}\frac{\omega\varepsilon_{r''}}{\gamma_{e}}|\mathbf{E}|^{2}+\frac{\mu_{0}}{2}\frac{\omega\mu_{r''}}{\gamma_{m}}|\mathbf{H}|^{2}$ (29)

It is worthwhile mentioning that this dissipative energy density does not go to zero as $\gamma$ does (it is logical since we did not calculate this limit). We can define an average dissipative energy density $\bar{w}_{d}$ as the product of the cycle-averaged dissipative power density ($\varepsilon_{0}\varepsilon''\omega|E|^{2}+\mu_{0}\mu''\omega|H|^{2}$) and the period of a cycle:

$\bar{w}_{d}=\frac{1}{2}\varepsilon_{0}\varepsilon''|\mathbf{E}|^{2}+\frac{1}{2}\mu_{0}\mu''|\mathbf{H}|^{2}$ (30)

where it clearly vanishes when $\varepsilon''$ and $\mu''$ goes to zero.

Therefore, inside dispersive and dissipative NZI materials materials, we can control the stored energy density via the real parts of $\varepsilon$ and $\mu$ while the dissipative energy density is controlled by the imaginary parts. The NZI materials limits lead to an absence of stored energy, which is fully consistent with the absence of momentum transfer inside NZI materials. Only dissipative energy survives. If losses are small, one retrieves our previous case of zero energy density in the NZI materials limit [6]

Ruppin gives an energy velocity equals to

$v_{E}=\frac{2cRe(\sqrt{\frac{\varepsilon}{\mu}})}{(\varepsilon'+\omega\frac{d\varepsilon'}{d\omega}) +(\mu'+\omega\frac{d\mu'}{d\omega})}.$ (31)

In the EMNZ limit (dispersive and dissipative), we get

$v_{E}=\frac{2cRe(\sqrt{\frac{\varepsilon}{\mu}})}{\omega(\frac{d\varepsilon}{d\omega}+\frac{d\mu}{d\omega}|\frac{\varepsilon}{\mu}|)}$ (32)

which leads, in case of a low loss limit to

$v_{E}=\frac{c}{\omega\frac{dn}{d\omega}}=v_{g}.$ (33)

## 2 Balazs gedanken experiment

First, let us discuss Balazs gedanken experiment [Balazs1953], well described in [MansuripurSPIE2011,BarnettLoudon2010]. We can have the following reasonging either with a short light pulse or using a photon [MansuripurSPIE2011]. We choose here the photon version.

Let us consider a transparent dielectric slab of length $L$, having a group refractive index $n_{g}(\omega)$ (Figure 2). The slab can move without any friction along the $x$ axis and is supposed to be initially at rest ($v=0$). The photon propagates in the $x$ direction, enter the slab from the left facet and exits from the right. We suppose no losses due to absorption or scattering. It possess an energy $\hbar\omega$ and propagates at velocity $c$ outside the slab. If one applies energy conservation before the photon enters the slab, the system (i.e. the slab+the photon) has a total energy

$E_{tot}=E_{phot,vac}+E_{slab,vac}=\hbar\omega+Mc^{2}$ (34)

where $M$ stands for the mass of the slab. The subscript $vac$ indicates that the photon is within vacuum at that time. The aim of the present consideration is to find the electromagnetic momentum of the photon inside the slab. Upon each encounter with the facets, the photon can either get reflected at the interface or go through the slab. Consequently, there will be an infinite number of possible outcomes for the present gedanken experiment. As Mansuripur did [MansuripurSPIE2011], we will examine only three significant cases:

• The photon bounces back from the first facet;

• The photon goes through both facets successively, without any reflection at all;

• The photon enters the slab, bounds two times are the right and left facets successively then exists at the right facet.

The simplest possibility is the first situation where the photon is directly reflected backwards at the slab interface. Its vacuum momentum $\frac{\hbar\omega}{c}$ reverses its direction and following momentum conservation, the slab acquires a forward momentum of $2\frac{\hbar\omega}{c}$. Let us recall here that momentum conservation implies that, for any system not subjected to external forces, the momentum of the system (here slab+photon) will remain constant. It means that the center of mass, or more precisely the centre of mass-energy, moves with a constant velocity [Einstein1906]. In this first case, the center of mass-energy of the system continues to move forward at the same rate as if the photon was travelling outside the slab (path 2). It is impossible to learn anything about the EM momentum inside the medium since the photon never entered the slab.

The second case is much more appealing since the photon propagates inside the slab over a distance $L$. Once it enters the medium, its speed slows down to $c/n_{g}(\omega)$ since, if there are no losses, the energy velocity is the group velocity $v_{g}(\omega)$ (See Appendix 5). It consequently takes the photon a time $\Delta t=n_{g}(\omega)L/c$ to travel through the medium. Therefore, when the photon emerges from the slab following path 1, it will be delayed due to this reduced velocity compared to a photon travelling on path 2, outside the slab. In other words, a photon following path 2 is ahead a distance $( n_{g}(\omega)-1) L=( \frac{c}{n_{g}(\omega)}-1) L$ compared to the photon emerging from the slab on path 1. The deviation from uniform motion can consequently only be made up if the block itself is displaced in the direction of propagation of the photon by an amount $\Delta x$ while the photon is in the medium. This displacement can be calculated as follows. The delay has caused a leftward shift of the product of mass by displacement to the photon:

$\frac{E_{phot,vac}}{c^{2}}\left( n_{g}(\omega)-1) \right)L=\frac{\hbar\omega}{c^{2}}\left( n_{g}(\omega)-1) \right)L$ (35)

where $\frac{\hbar\omega}{c^{2}}$ is the corresponding mass of the photon. This shift must be compensated by a rightward shift of the slab itself:

$\Delta xM.$ (36)

Equating both leftward and rightward mass times displacements yields

$\Delta x=\left( n_{g}(\omega)-1) \right)\frac{\hbar\omega L}{Mc^{2}}.$ (37)

We can clearly observe that this displacement depends linearly on the thickness of the slab, the ratio of the photon and material energies and the group refractive index. If the medium would have been made of vacuum, $\Delta x=0$. Consequently, the slab acquired a momentum from the photon in order to move the distance $\Delta x$:

$p_{slab}=Mv=M\frac{\Delta x}{\Delta t}=\left( 1-\frac{1}{n_{g}(\omega)} \right)\frac{\hbar\omega}{c}.$ (38)

Now, by simply applying momentum conservation to the system, we deduce the momentum of the photon inside the slab $p_{phot,slab}$:

$p_{phot,vac}+p_{slab,vac}=p_{phot,slab}+p_{slab,slab}$ (39)

and considering that the slab is initially at rest, it leads to

$p_{phot,slab}=\frac{\hbar\omega}{cn_{g}(\omega)}=p_{A}.$ (40)

We see that the momentum of the photon inside the slab is nothing but Abraham momentum.

The third case can be treated in a similar way, reaching the same conclusion for the EM momentum. If the photon enters the slab from the left, bounces back two times at the facets then exists on the right facet, it will have spent a total time of $\Delta t'=3n_{g}(\omega)L/c$ seconds inside the slab. Its delay will be $\left( 3n_{g}(\omega)-1 \right)L\frac{\hbar\omega}{c^{2}}$ compared to free-space photon on path number 2. Equating the leftward and rightward mass times displacement of both the photon and the slab yields $p_{phot,slab}=\frac{\hbar\omega}{cn_{g}(\omega)}$ in any cases for the EM momentum inside the dielectric.

## 3 Impact of losses on momentum considerations

Any real, physical metamaterial or photonic crystal used to produce NZRI characteristics has to be dispersive and present some degree of losses. Specifically, metal oxide films (ITO or Al-doped Zn oxide for example) or metal-dielectric (hyperbolic) metamaterials are systems exhibiting NZRI index but large optical losses and high impedance. Those drawbacks are detrimental to applications. It therefore motivated an all-dielectric photonic crystal road in order to eliminate metallic dissipative losses (e.g. see [7,8]).

Although material loss in an all-dielectric photonic crystal is negligible, the zero index modes are above the light-line causing substantial radiative losses both in- and out-of-plane. Nevertheless, in-plane radiative losses can be mitigated using photonic bandgap structures while out-of-plane ones need novel strategies such as bound states in the continuum approaches. This leads to high quality factors NZRI photonic crystals that could operate to a broad range of visible to infrared frequencies.

Nevertheless, in the lossless limit, the dispersion locally becomes flat, but the second order derivative is non-zero. Accordingly, there is dispersion. EMNZ will have dispersion and a nonzero group velocity, even if lossless.

We can quantify the above statement by supposing a Lorentz model for both electric permittivity and magnetic permeability, i.e. $\varepsilon\left( \omega\right)=\mu\left( \omega\right)=\frac{\omega^{2}-\omega_{Z}^{2}+2i\omega\Gamma}{\omega^{2}-\omega_{r}^{2}+2i\omega\Gamma}$ with $\omega_{r}=0.1\omega_{Z}$. We can then check the influence of the losses, represented by the $\Gamma$ factor, on the presented formalism. The following values are chosen $\Gamma=1\times{10}^{-3}\omega_{Z}$, $\Gamma=1.5\times{10}^{-2}\omega_{Z}$ and $\Gamma=1\times{10}^{-1}\omega_{Z}$. It leads to $\varepsilon\left( \omega_{Z} \right)=\mu\left( \omega_{Z} \right)=0.002i$,$\varepsilon\left( \omega_{Z} \right)=\mu\left( \omega_{Z} \right)=0.03i$ and $\varepsilon\left( \omega_{Z} \right)=\mu\left( \omega_{Z} \right)=0.039+0.19i$ respectively. We should note that the second $\Gamma$ corresponds to losses inside SiC. The impact of those realistic losses is shown below. Figure S1 represents real and imaginary parts of electric permittivity and magnetic permeability of the EMNZ material. Crossing appears at $\omega=\omega_{Z}$, except for $\Gamma=1\times{10}^{-1}$ where there is a slight offset. Figure S2 provides the corresponding group index.


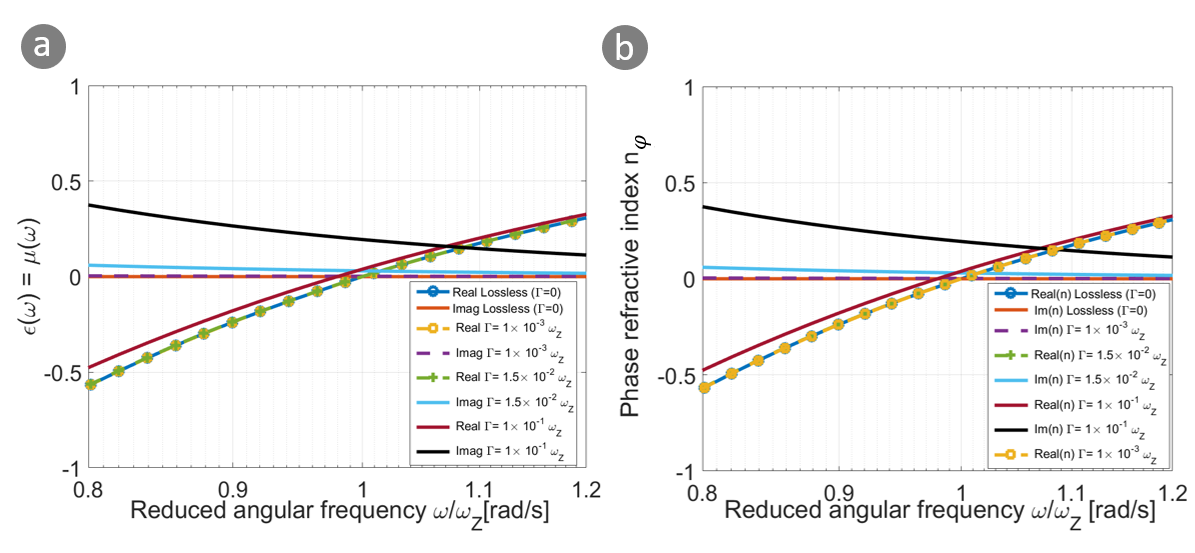


Figure S1. (a) Lorentz model for electric permittivity $\varepsilon(\omega)$ and magnetic permeability $\mu(\omega)$ with $\omega_{r}=0.1\omega_{Z}$ and (b) corresponding phase refractive index $n_{\varphi}\left( \omega\right)$ around the zero-index frequency $\omega_{Z}$ for different losses $\Gamma$.


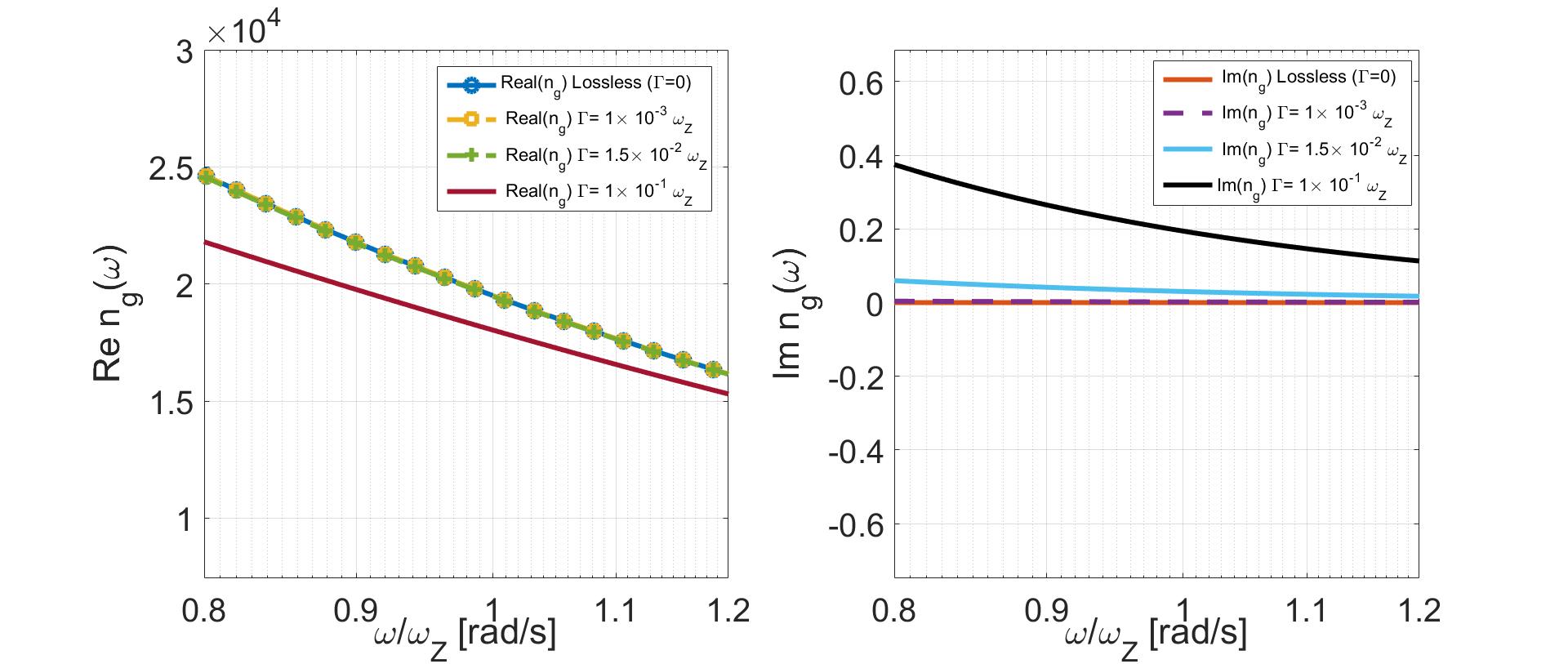


Figure S2. Corresponding group refractive index $n_{g}\left( \omega\right)$ around the zero-index frequency $\omega_{Z}$ for different losses $\Gamma$.


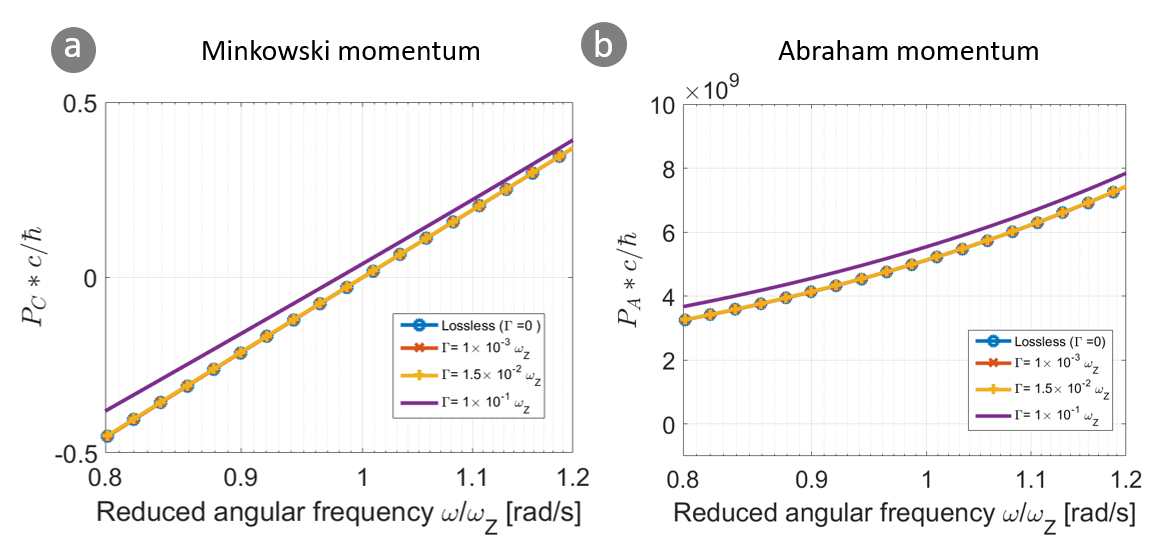


Figure S3. Minkowski/canonical and Abraham momenta around the zero-index frequency $\omega_{Z}$ for different losses $\Gamma$.

As we can see on figure S3, the canonical momentum inside EMNZ material crosses zero at $\omega=\omega_{Z}$ and the impact of losses is moderate. Similarly, the Abraham momentum inside EMNZ had a nonzero value but the impact of losses is not critical.

A similar discussion can be done numerically using COMSOL for discerning the impact of losses on diffraction (Figure 4 main text). As we can see on Figure S4, the pattern and the positions of the minima/maxima of diffraction do not change with losses, only the intensity gets reduced.


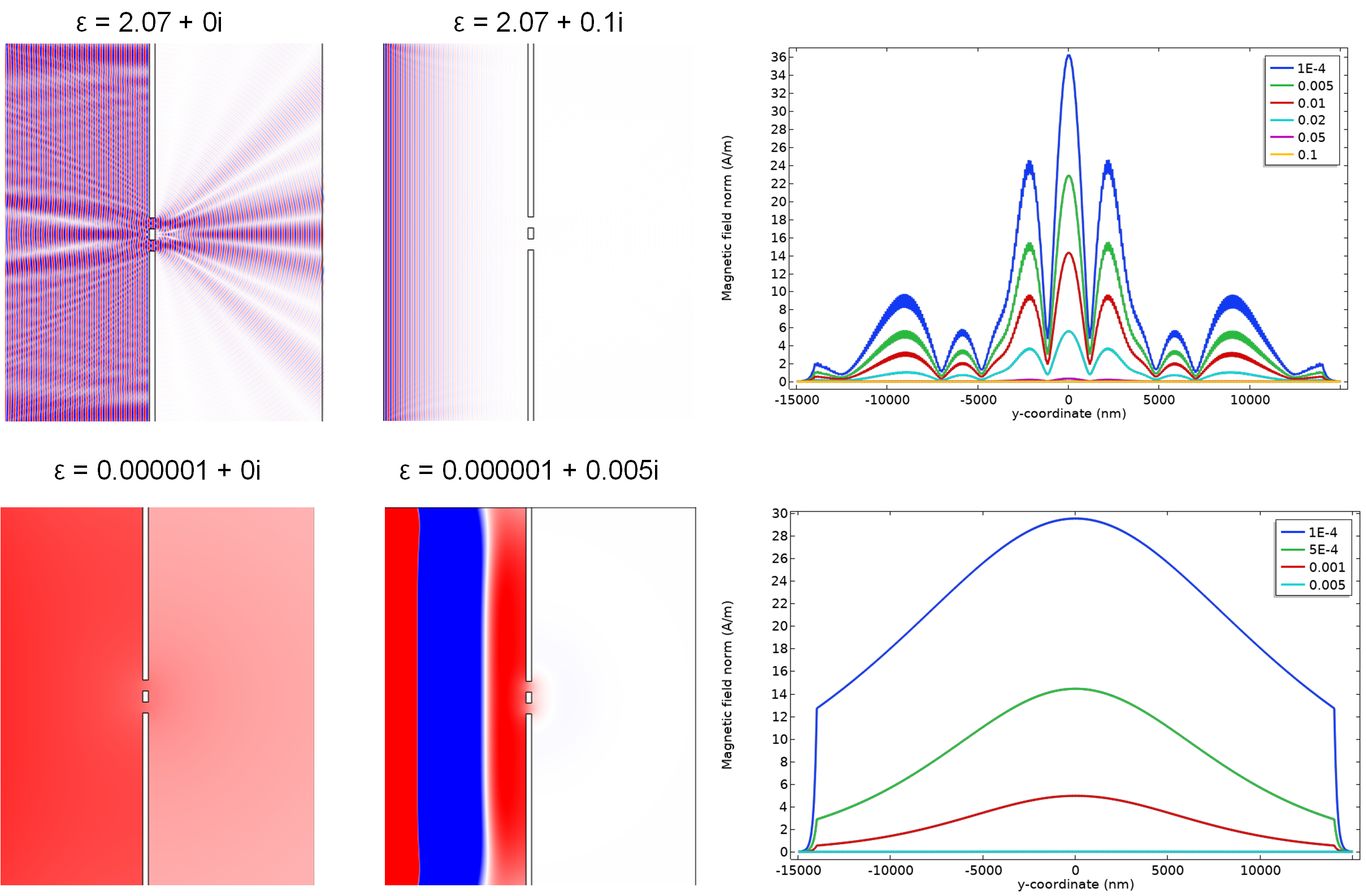


Figure S4: Young double-slit experiment within different dispersive materials with different amount of losses. Left: $|H_{z}|$ field maps, right: $|H|$ profile on the observing screen.

**References**

[1] Rodney Loudon,*The quantum theory of light*,Oxford University Press (2000).

[2] Frederico Dias Nunes, Thiago Campos Vasconcelos, Marcel Bezerra, John Weiner, and Grupo De Engenharia, Electromagnetic energy density in dispersive and dissipative media,  *JOSA B*, 28,1544–1552 (2011).

[3] R Ruppin, Electromagnetic energy density in a dispersive and absorptive material,  *Phys. Lett. A*, 299,309–312 (2002).

[4] Shivanand and Kevin J. Webb, Electromagnetic field energy density in homogeneous negative index materials, *Opt. Express*, 20,11370–11381 (2012).

[5] Equivalent relations can be derived for permeability.

[6] Michael Lobet, Iñigo Liberal, Erik N Knall, M Zahirul Alam, Orad Reshef, Robert W Boyd, Nader Engheta, and Eric Mazur, Fundamental radiative processes in near-zero-index media of various dimensionalities, *ACS Photonics*, 7, 1965-1970 (2020).

[7] H. Tang et al., Nano Letters 2021 21 (2), 914-920

[8] Larissa Vertchenko, Clayton DeVault, Radu Malureanu, Eric Mazur,

and Andrei Lavrinenko, Near-Zero Index Photonic Crystals with Directive Bound

States in the Continuum, Laser Photonics Rev. 2021, 2000559.
